# Transcriptome Reveals Regulation of Quorum Sensing of *Hafnia alvei* H4 on the Coculture System of *Hafnia alvei* H4 and *Pseudomonas fluorescens* ATCC13525

**DOI:** 10.3390/foods13020336

**Published:** 2024-01-21

**Authors:** Yanan Wang, Xue Li, Gongliang Zhang, Jingran Bi, Hongman Hou

**Affiliations:** 1 School of Food Science and Technology, Dalian Polytechnic University, No. 1 Qinggongyuan, Ganjingzi District, Dalian 116034, China; yananwang0226@163.com (Y.W.); lixuexiaomuzhu@163.com (X.L.); zgl_mp@163.com (G.Z.); bijr@dlpu.edu.cn (J.B.); 2 Liaoning Key Lab for Aquatic Processing Quality and Safety, No. 1 Qinggongyuan, Ganjingzi District, Dalian 116034, China

**Keywords:** *Hafnia alvei* H4, *Pseudomonas fluorescens*, cocultivation, QS regulation, transcriptome

## Abstract

In the food industry, foodborne spoilage bacteria often live in mixed species and attach to each other, leading to changes in spoilage characteristics. Quorum sensing (QS) has been reported to be a regulating mechanism for food spoiling by certain kinds of bacteria. Here, the contents of biofilm, extracellular polysaccharides, and biogenic amines in the coculture system of *Hafnia alvei* H4 and *Pseudomonas fluorescens* ATCC13525 were significantly reduced when the QS element of *H. alvei* H4 was deleted, confirming that QS of *H. alvei* H4 is involved in the dual-species interactions. Then, transcriptomics was used to explore the regulatory mechanism at the mRNA molecular level. The deletion of the QS element decreased the transcript levels of genes related to chemotaxis, flagellar assembly, and the two-component system pathway of *H. alvei* H4 in the coculture system. Furthermore, a total of 732 DEGs of *P. fluorescens* ATCC13525 were regulated in the dual species, which were primarily concerned with biofilm formation, ATP-binding cassette transporters, and amino acid metabolism. Taken together, the absence of the QS element of *H. alvei* H4 weakened the mutual cooperation of the two bacteria in the coculture system, making it a good target for managing infection with *H. alvei* and *P. fluorescens*.

## 1. Introduction

Aquatic foods are highly perishable due to their high protein content [1], in which microorganisms are necessary for food spoilage because of their ability to degrade nutrients [2]. The development and metabolic processes of microorganisms produce sulfides, amines, alcohols, ketones, aldehydes, and organic acids, which have disagreeable and undesirable odors [3]. But only a limited number of microorganisms, known as special spoilage microorganisms (SSOs), can defeat other microbes, grow significantly, and generate the appropriate metabolites [4,5].

*Hafnia alvei* was identified as the SSO of vacuum-packed spoiled meat [6] and spoiled seafood [7]. Furthermore, research has demonstrated that *H. alvei* can mediate the expression of various spoilage enzymes [8]. Amino acid decarboxylases, for instance, convert amino acid precursors into corresponding biogenic amines in *H. alvei*, which accelerates its spoilage [9,10]. *Pseudomonas fluorescens* is a common parasite in fish [11], poultry [12], dairy [13], and other fresh products. *P. fluorescens* is well-known for being able to generate dense biofilms [14,15], which contributes to the risk of food contamination with spoilage [16,17]. It has been discovered that the two most common Gram-negative bacteria found in damaged milk and chill-stored proteinaceous raw foods are *P. fluorescens* and *H. alvei* [18,19]. This suggests that these two organisms pose a threat to the food industry and food safety, and further research is urgently needed to control contamination from these bacteria to slow down the deterioration of the quality of fresh food. Naturally spoiled food contains numerous bacterial species and strains. The microbial interactions that occur during meat spoilage have a significant impact on the microbiota composition. Spoilage characteristics can be influenced by various relationships, including antagonism, mutualism, and commensalism, among the microbiota members [20]. Therefore, the study of the interaction between bacteria under coculture conditions is more in line with the real natural environment of food.

QS is a density-dependent mechanism that regulates gene expression in bacteria by secreting and detecting small molecules called autoinducers. The N-acyl-1-homoserine lactone (AHL) synthase and AHL reporter are involved in the QS system, which plays an important role in regulating biofilm formation and the expression of spoilage genes. This demonstrates the relationship between the QS system, biofilm formation, and the potential for spoilage [21]. A previous study has indicated that in *H. alvei* H4, there is a LuxI/R-type QS system present. Additionally, there was no AHL synthesis in the *luxI* mutant [7], indicating that quorum sensing is disrupted. However, the biological properties and importance of these systems have mainly focused on mono-species cultures with reasonably well-established conditions [22], where most microorganisms live in complex communities [23]. QS signals can be used as a strategy for bacteria to compete with other microbes coresiding in the same environment [24]. The overall role of QS as a regulator of interspecies interactions with mixed species is relatively poorly understood, especially in the context of QS-regulated effectors. *H. alvei* induces other bacterial species in the same environment to display food-quality-related behaviors [6], but the mechanism is not understood. Therefore, it is particularly necessary to explore the mechanism of interaction and QS between *H. alvei* H4 and other spoilage bacteria.

In this study, the effect of QS of *H. alvei* H4 WT on biofilm development, extracellular polysaccharide, and biogenic amine production in both *H. alvei* H4 and *P. fluorescens* ATCC13525 was assessed. Subsequently, dual-seq analyses were performed on the cells to uncover the regulatory mechanism of QS in regulating the spoilage ability of the two bacteria.

## 2. Materials and Methods

### 2.1. Bacterial Strains and Growth Conditions

The wild-type *H. alvei* H4 (*H. alvei* H4 WT) and a *luxI* mutant (*H. alvei* H4 Δ*luxI*), which had been previously constructed in our lab, were used in this study [7]; *P. fluorescens* ATCC13525 was purchased from the BeNa Culture Collection Centre. Every strain was regularly cultured in Luria Bertani (LB) medium (10 g/L tryptone, 10 g/L sodium chloride, and 5 g/L yeast extract) at 30 °C, and 20 μg/mL chloramphenicol was added for ∆*luxI* incubation.

### 2.2. Crystal Violet Biofilm Assay

The biofilms generated by *P. fluorescens* ATCC13525 and *H. alvei* H4 WT/Δ*luxI* single or dual species were compared using a crystal violet (CV) assay, in accordance with the method provided by Hou et al. [25]. The overnight-cultured *P. fluorescens* ATCC13525 and *H. alvei* H4 cultures were diluted 100-fold into 96-well plates (about 10^5^ CFU/mL). After 12, 24, 36, and 48 h of incubation at 30 °C under static conditions, the culture medium was carefully collected and cleaned with sterile PBS (pH 7.0). Subsequently, 250 µL of anhydrous methanol was added and incubated for 15 min to preserve the cells. After staining with CV (0.1% *w*/*v*) for 15 min, the biofilm cells that clung to the lower surface and sides of each well were removed using deionized water (250 µL per well). The CV was resolubilized with 200 µL of 33% glacial acetic acid and then shaken at a speed of 300 rpm for a duration of 15 min. The optical density of the solution was subsequently measured at OD_590_ nm using a Spectra M2 spectrophotometer.

### 2.3. Extracellular Polysaccharides Assay

The procedure described by Harimawan et al. [26] was followed to extract extracellular polysaccharides of biofilm. Briefly, at each time interval (12, 24, 36, and 48 h), 10 mL of cultures was carefully aspirated and then centrifuged at 4 °C (8000× *g*, 15 min). Then, the precipitate was resuspended in saline, and it was placed in a water bath set at 80 °C for 30 min. After a 30 min centrifugation at 4 °C (12,000× *g*), the supernatant was passed through a 0.22 µm membrane. The content of exopolysaccharides in the supernatant was measured using the phenol sulfuric acid method.

### 2.4. Biogenic Amine Measurement Using HPLC

The amount of putrescine or cadaverine was measured using the previously described method [27]. The single colonies of three strains were selected and cultured in the amino acid medium at 30 °C and 150 rpm of shaking until the medium became turbid, and then, it was transferred again. At each time interval (12, 24, 48, and 72 h), 1 mL of cultures was carefully aspirated, 9 mL of 10% trichloroacetic acid (TCA) was added, and it was stored for 2 h at 4 °C. Subsequently, centrifuged at 4 °C (3000× *g*, 10 min) for 10 min, 200 µL of the liquid above the solid residue was pipetted, and 80 µL NaOH (2M), 120 µL of saturated NaHCO_3_, and 800 µL of dansyl chloride (10 mg/mL, prepared in acetone) were added in order to complete the derivatization process. Following that, the mixture was placed in a warm bath set at 45 °C for 40 min. Subsequently, 50 µL of ammonia was added, and the mixture was allowed to sit at room temperature for 30 min. Finally, 550 µL of chromatography grade acetonitrile was added and centrifuged at 4 °C for 5 min at 3000× *g*. The resulting supernatant was filtered three times through a 0.22 µm filter membrane and stored at −80 °C. The same procedure was followed to detect biogenic amines produced by *H. alvei* H4 and Δ*luxI* in the presence of *P. fluorescens* ATCC1355.

The amount of BA was measured using an Agilent 1260 HPLC unit (Agilent Technologies Inc., Santa Clara, CA, USA) with an Agilent Zorbax SB-C 18 column (4.6 × 150 mm). There was a 1.0 mL/min flow rate. The column was eluted using an optimized gradient comprising ultrapure water (Solvent A) and acetonitrile (Solvent B) in a binary solution, following an optimized gradient: 0–10 min, 55% B; 10–15 min, 55–65% B; 15–20 min, 65–80% B; 20–25 min, 80% B; 25–30 min, 80–90% B; 30–33 min, 90% B; 33–35 min, 90–55% B.

### 2.5. Transcriptome Analysis and Quantitative Reverse Transcription (RT-qPCR)

The method of transcriptome analysis was referenced from Xue et al. [28]. *H. alvei* H4 and *P. fluorescens* ATCC13525 in dual species were used to extract RNA using the RNA prep pure Cell/Bacteria Kit (Tiangen Biotech, Beijing, China). Before conducting the experiments, the mRNA was isolated from total RNA by employing specific probes to eliminate rRNA contaminants. The fragmentation process was then carried out by subjecting the mRNA to high temperatures in the presence of divalent cations in the First Strand Synthesis Reaction Buffer (5X). For the generation of the first strand cDNA, a random hexamer primer was utilized along with M-MuLV Reverse Transcriptase (RNase H-). Subsequently, in the DNA polymerase I system, the dUTP was used as a substitute for dTTP in order to synthesize the second strand of cDNA. Finally, to ensure the selection of cDNA fragments in the desired length range of 370~420 bp, the library fragments underwent purification using the AMPure XP system from Beckman Coulter in the USA. PCR was then performed with Phusion High-Fidelity DNA polymerase, Universal PCR primers and Index (X) Primer. At last, the Agilent Bioanalyzer 2100 System was used to evaluate the library quality after the PCR products had been purified using the AMPure XP System.

RT-qPCR experiments with SYBR Green were performed to validate genes of interest and specific genes associated with the spoilage factors or viability of *H. alvei* H4 and *P. fluorescens* ATCC13525. Threshold cycles for the endogenous control (16S rRNA) were used to standardize the data. The cycle number at which the intensity of the fluorescence reached a predefined threshold was identified as the threshold cycle or Ct. Each gene was quantitated using the −2^−ΔΔCt^ method.

### 2.6. Statistical Analysis

Every experiment was repeated in triplicate. The statistical software IBM SPSS (v19.0, IBM SPSS Inc., Armonk, NY, USA) was used to perform a one-way analysis of variance (ANOVA) on the data, which were reported as mean ± standard deviation (SD). A portion of the data was examined on the online platform of NovoMagic Cloud Platform https://magic.novogene.com (accessed on 16 August 2023).

### 2.7. Accession Numbers

The raw and processed transcriptome data of *H. alvei* H4 and *P. fluorescens* ATCC13525 in dual RNA seq were deposited at the Gene Expression Omnibus (GEO) database under the accession number GSE245811.

## 3. Results and Discussion

### 3.1. Characteristics under Coculture Conditions

#### 3.1.1. Biofilm Formation

**Figure 1:**
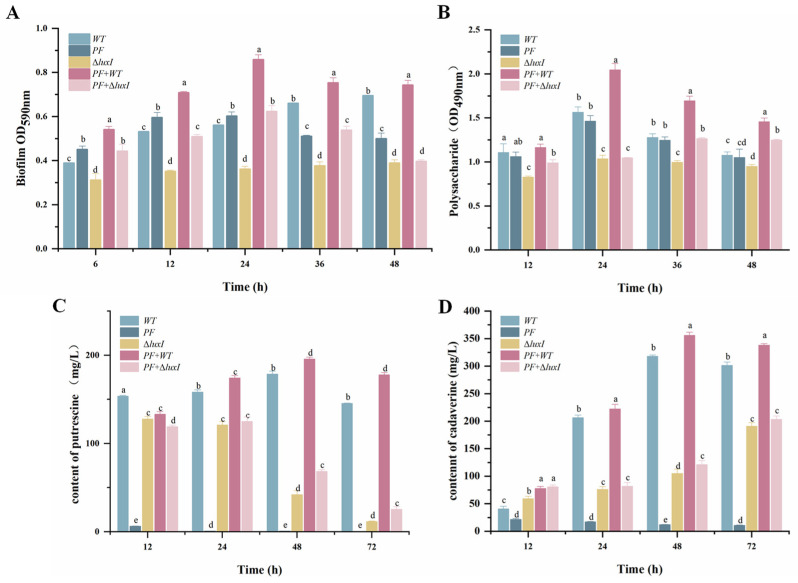
Changes in spoilage phenotypes in monoculture and coculture systems at different times. (**A**) Biofilm biomass; (**B**) polysaccharide; (**C**) putrescine; (**D**) cadaverine. WT: *Hafnia alvei* H4 wild type; PF: *Pseudomonas fluorescens* ATCC13525; Δ*luxI*: *Hafnia alvei* H4 *luxI* mutant strain; PF + WT: *P. fluorescens* ATCC13525 cocultured with *H. alvei* H4; PF + Δ*luxI*: *P. fluorescens* ATCC13525 cocultured with *H. alvei* H4 Δ*luxI* a, b, c, d and e represent significant differences between groups.

In the food industry, bacteria can adhere to solid surfaces to form biofilms, which are extremely difficult to detect and eradicate [29]. In order to investigate whether biofilm formation is influenced by interspecies interactions between *H. alvei* H4 and *P. fluorescens* ATCC1355, crystal violet staining was used to monitor the biofilm formation of *H. alvei* H4 and *P. fluorescens* ATCC1355 in a mono-inoculated or co-inoculated system in LB medium. First, there was a synergistic interaction between *H. alvei* H4 and *P. fluorescens* ATCC1355, where an increase in optical density was observed for the dual-species biofilms compared with biofilms formed by a single-culture strain (Figure 1A). Consistent with previous reports, mixed species always showed a high potential for biofilm formation, both under natural conditions and in the laboratory environment [30]. The hypothesis is that the synergistic effect in dual-species biofilms could be attributed to QS of *H. alvei* H4 WT. In particular, reductions in dual-species biofilm formation were observed when the *luxI* gene was deleted. These findings suggest that QS of *H. alvei* H4 WT plays a favorable role in the development of biofilms involving two species (Figure 1A). It may be that the destruction of QS led to the weakening of other metabolic functions in *H. alvei* H4 WT, which, in turn, affected the activities of *H. alvei* H4 WT and the cocultured *P. fluorescens* ATCC13525.

#### 3.1.2. Exopolysaccharide Assay

Polysaccharides are also major components of EPS in the *P. fluorescens* biofilm matrix [14]. The amount of polysaccharides in mono- and dual-species biofilms was quantified, as presented in Figure 1B. Similar results to the optical density biofilm assay were obtained for the exopolysaccharide secretion. All strains demonstrated a strong exopolysaccharide-forming ability at 30 °C, especially the wild-type coculture system, whose exopolysaccharide biomass was significantly higher than that of the single species, indicating that the interactions between *H. alvei* H4 WT and *P. fluorescens* ATCC13525 promoted the capacity of the biofilm. However, the EPS secretion viability of *P. fluorescens* ATCC13525 cocultured with *H. alvei* Δ*luxI* was significantly decreased, suggesting the critical role of QS in the regulation of the interaction between *H. alvei* H4 WT and *P. fluorescens* ACTT13525.

#### 3.1.3. Biogenic Amine Production

The formation of BAs is a result of amino acid decarboxylation, which is associated with the activities of microorganisms [31]. Putrescine (PUT) and cadaverine (CAD) are two common BAs found in spoiled seafood, with putrescine being synthesized through ornithine decarboxylation or agmatine deamination and cadaverine originating from lysine decarboxylation [32]. The content of PUT and CAD in various culture systems was measured to investigate the impact of QS on these biogenic amines (Figure 1C,D). It was demonstrated that at each period, the amount of PUT and CAD produced by *H. alvei* H4 WT was higher than *P. fluorescens* ACTT13525, which was almost undetectable. The amount of *H. alvei* H4 Δ*luxI* biogenic amines was substantially reduced (*p* < 0.05) compared with the WT strain, and when they combined with *P. fluorescens* ACTT13525, the wild-type coculture system was still at an advantage in terms of yield. The higher levels of biogenic amines found in the dual species could be attributed to the influence of QS on the production of amino acid decarboxylase.

### 3.2. Transcriptome Analysis under Coculture Conditions

#### 3.2.1. Transcriptional Responses in *H. alvei* H4 and *P. fluorescens* ATCC13525

**Figure 2:**
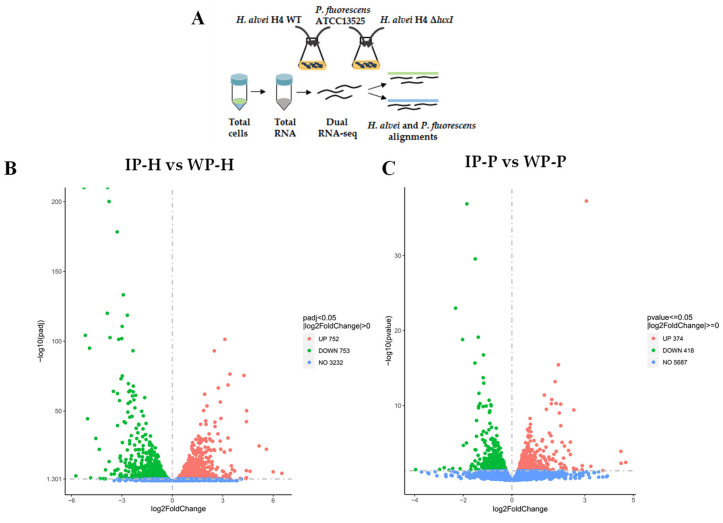
Experimental setup and total analysis of differentially expressed genes in *Hafnia alvei* H4 and *Pseudomonas fluorescens* ATCC13525. (**A**) Experimental setup for culture preparation and RNA extraction. (**B**) Volcano plot displaying differential regulation of *H. alvei* H4 genes in IP-H vs. WP-H; (**C**) Volcano plot displaying differential regulation of *P. fluorescens* ATCC13525 genes in IP-P vs. WP-P. Green and red dots represent down- and upregulated genes, respectively, and blue dots represent genes whose expressions were not altered significantly.

The cocultures formed by duo species (*H. alvei* H4 WT + *P. fluorescens* ACTT13525) and duo species (*H. alvei* H4 Δ*luxI* + *P. fluorescens* ACTT13525) underwent RNA-seq. Sequences read from two different transcriptomes were mapped to the reference genomes of two bacteria (*H. alvei* and *P. fluorescens*), respectively, using bioinformatic methods (Figure 2A). Among these wild-type coculture system biological replicates, an average of 55.38% clean reads were mapped to the *H. alvei* reference genome (data set WP-H), and 43.93% clean reads were mapped to the *P. fluorescens* ATCC13525 reference genome (data set WP-P). Among these deletion-type coculture systematic biological replicates, an average of 50.52% clean reads were mapped to the *H. alvei* reference genome (data set IP-H), and 48.73% clean reads were mapped to the *P. fluorescens* ATCC13525 reference genome (data set IP-P).

**Figure 3:**
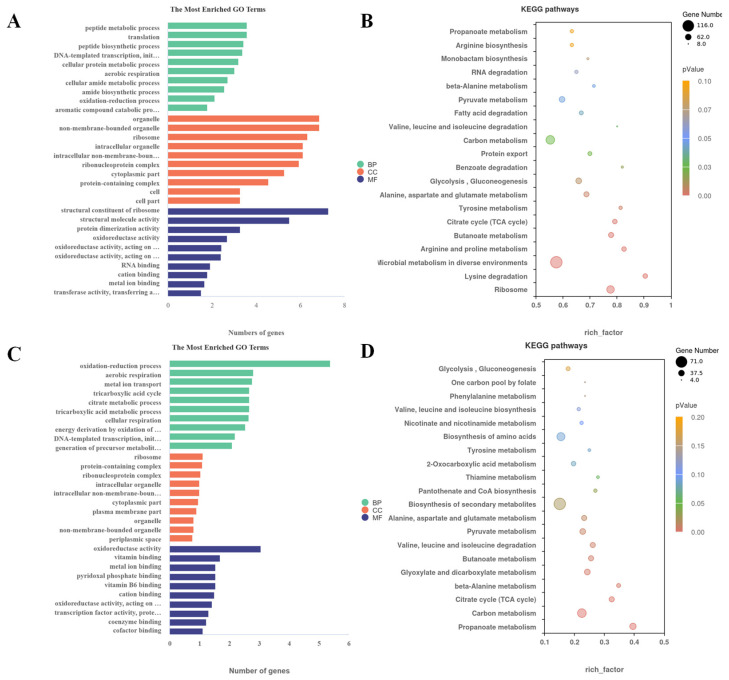
Statistical enrichment of DEGs in GO and KEGG pathways (MF: molecular function, BP: biological process, CC: cellular component). (**A**) GO terms of DEGs in *Hafnia alvei* H4 of IP-H vs. WP-H; (**B**) Scatter diagram of the top 20 KEGG enrichment pathways for DEGs in *H. alvei* H4 of IP-H vs. WP-H; (**C**) GO terms of DEGs in *Pseudomonas fluorescens* ATCC13525 of IP-P vs. WP-P; (**D**) Scatter diagram of the top 20 KEGG enrichment pathways for DEGs in *P. fluorescens* ATCC13525 of IP-P vs. WP-P.

In the differential gene expression analysis of RNA-seq data, *H. alvei* H4 and *P. fluorescens* ATCC13525 in the wild-type coculture system were used as controls. In *H. alvei* H4 Δ*luxI*, 1428 of the identified genes were significantly differentially expressed (DEGs), of which 675 were upregulated, and 753 were downregulated by comparing IP-H and WP-H. Similarly, a total of 792 DEGs were detected in *P. fluorescens* ACTT13525 in IP-P, including 418 upregulated and 374 downregulated DEGs. Volcano plots were used to visualize the distinct transcriptional profiles of DEGs in the two comparisons (Figure 2). To obtain a more profound understanding of the potential functional differences resulting from DEGs, a comprehensive functional grouping analysis based on the GO and KEGG database mapping was carried out (Figure 3). The enrichment genes in the three GO categories are summarized in Figure 3; all DEGs were categorized into three distinct groups: biological processes, cellular components, and molecular functions. The most enriched terms in these three categories in *H. alvei* H4 were the peptide metabolic process, organelles, and the structural constituents of ribosomes, respectively (Figure 3A). In *P. fluorescens* ATCC13525, after being cocultured with *H. alvei* H4 Δ*luxI*, the oxidation–reduction process, ribosome, and oxidoreductase activity were the most enriched terms in these three categories, respectively (Figure 3C). The KEGG database was used to further analyze the biological functions related to DEGs, and the results reveal 20 significantly changed metabolic pathways (Figure 3).

Many differential genes are involved in (but not limited to) flagellar assembly, bacterial chemotaxis, transcriptional regulators, the signal transduction system, and amino acids metabolism (Table 1 and Table 2). The following is a detailed discussion of the KEGG enrichment pathways that are engaged in the reaction network.

#### 3.2.2. Analysis of Genes Associated with Biofilm Formation

##### Flagellar Assembly and Bacterial Chemotaxis

Bacterial attachment to the surface is an essential initial step in biofilm formation, which is facilitated by flagella. It has been suggested that the flagellum is responsible for motility in Gram-negative bacteria [33]. The primary components of flagellar are the matrix, hook, and filaments. As shown in Table 1, gene downregulation caused by QS disruption was mainly focused on flagellar structural genes (*flgBCDE*, *flgFGHIJKL*, *fliCfliDfliS*, fliJK, *fliMN*, and *flhA*), two transcriptional regulatory protein genes (*flgM* and *fliA*), and several genes encoding chemotaxis proteins (*motA* and *cheW*) in *H. alvei* H4 Δ*luxI* (Table 1). The flagellar basal-body protein and lateral flagellar protein are encoded by the *flgC* and *flgL* genes. FlgD and FliN are important components of the flagellar hook and the flagellar cytoplasmic C-ring (the mechanical core of the flagella), respectively, while MotA is a motility protein present on the surface of the bacteria that provides the power for normal flagellar motility [34,35,36]. The downregulation of the above genes may reduce the fixation ability and driving ability of the substrate, ultimately leading to a decrease in bacterial adhesion.

In line with these observations, the downregulated genes of *P. fluorescens* ATCC13525 in response to QS of *H. alvei* H4 WT were two genes encoding transcription regulators and RNA polymerase sigma factor, including *flgM*, encoding the anti-σ-28 factor, which plays a crucial role in regulating the flagellar number and flagellar assembly rate, and *fliA* (as a σ factor regulating bacterial flagella gene expression) [37]. These results indicate that the downregulation of *flgM* and *fliA* in the IP-P group was due to the absence of the QS element in *H. alvei* H4 WT and further indicated that QS of *H. alvei* H4 WT exerted a positive effect on the flagellar synthesis transcriptional regulators of *P. fluorescens* ATCC13525, rather than directly on the basic structure of the flagellum.

Bacterial chemotaxis is a crucial mechanism in various biological processes, such as the formation of biofilms and the regulation of quorum sensing, etc. It relies on the mobility of bacteria, wherein their movement is triggered by chemical stimuli [38]. The entire process of bacterial chemotaxis is governed by a complex interplay among different proteins, including methyl-accepting chemotactic proteins (MCPs) and cytoplasmic proteins (Ches). MCPs are responsible for sensing signals, while cytoplasmic proteins process sensory signals and then transmit control signals to flagellar motors for chemotactic movement [39]. MCPs are able to cross the cell membrane, detect chemical changes in the surrounding environment, and then send out signals by themselves to induce bacteria to adapt to growth. The downregulation of MCPs leads to the decreased ability of bacteria to sense signals in the environment [40]. In addition, a previous study showed that the phosphorylated form of cytoplasmic proteins cheY binds to FliM and FliN, switching the rotation direction of the rotation of flagellar motors [41]. In this study, the expression of *tsr* (encoding MCPs) was downregulated in the IP-H group, and the genes encoding FliM and FliN were also significantly downregulated (Table 1). Therefore, the loss of the QS element can lead to the inhibition of the expression of chemotactic-related genes in *H. alvei* H4 WT during coculture, further affecting the content of biofilms. In *P. fluorescens* ATCC13525, the chemotactic adaptation protein CheW, which interacts with MCPs and CheA and is necessary for the MCP-mediated control of CheA activity, was likewise downregulated [42]. This suggests that QS in *H. alvei* H4 WT influences the chemotactic expression of *P. fluorescens* ATCC13525 in a coculture system. The expression of these chemotactic genes in *H. alvei WT* and *P. fluorescens* is also regulated by the AHL-mediated QS system, which is consistent with the case in *Aeromonas* [43].

##### Signal Transduction System

Bacteria have multiple signal transduction systems, and two-component systems (TCSs) are significant signaling pathways that control chemotaxis, biofilm formation, and responses to environmental stimuli [44]. The primary constituents of the TCS are histidine protein kinases (HKs) and response regulators (RRs). The EnvZ/OmpR system is a common two-component system, which creates a viable way for bacteria to survive in adverse conditions [45]. Briefly, OmpR is activated by EnvZ and binds to the target gene promoter, activating the expression of corresponding genes such as *ompF* and *ompC*. Thereby, it senses external signal stimuli and modulates the motility of microbial cells, thus affecting biofilm formation [46]. As shown in Table 2, the downregulation of these signal transduction genes for *envZ* (encoding HKs) and *ompR* (encoding RRs) further proves that QS of *H. alvei* H4 WT may influence biofilm formation by modulating its own signaling in the coculture, which is similar to the result that DEGs enriched in TCSs may sense external signal stimuli and modulate the motility of microbial cells, thus influencing the formation of biofilms [46]. We found that in response to the destruction of QS, most of the DEGs related to signal transduction were involved in the two-component system of *H. alvei* H4 WT.

However, the genes for ABC transporters in *P. fluorescens* ATCC13525 were among the most strongly downregulated genes when cocultured with *H. alvei* H4 Δ*luxI*. ABC transporters play a crucial role in facilitating the transportation of essential nutrients and the removal of harmful substances across the membrane in microorganisms. This effectively enhances the microorganisms’ ability to resist stressful conditions [47]. Additionally, these transporters are responsible for carrying a diverse range of substrates, such as ions and macromolecules, as well as materials required for biofilm formation [48]. *RbsB* is a ribose-binding protein, a subset of which is also the primary chemoreceptor for chemotaxis. The research of Zhang et al. [49] suggests that *RbsB* may play an important role in QS systems, and Armbruster et al. [50] also demonstrate that the deletion of *RbsB* significantly affects the absorption of AI-2 and biofilm formation. In this study, the expression of *RbsB* in the IP-P group was downregulated (Table 2), which may explain the decrease in biofilm formation during the coculture of *P. fluorescens* ATCC13525 and *H. alvei* H4 Δ*luxI* (Figure 1A).

Bacteria can cooperate with each other to form dense biofilms on the surfaces of various food production equipment, which are difficult to remove and cause food spoilage. The above results demonstrate that disrupting QS of *H. alvei* H4 WT can downregulate genes related to biofilm formation in cocultures, thereby reducing biofilm formation (Figure 1), and is an effective way to inhibit food spoilage.

#### 3.2.3. Amino Acid Metabolism

BAs are organic compounds with biological activity that result from the decarboxylation of precursor amino acids caused by microorganisms’ amino acid decarboxylases [51]. Therefore, the contents of precursor amino acids are highly likely to affect the production of biogenic amines by microorganisms. According to our previous results, CAD and PUT are the main BAs found in *H. alvei* H4 WT. CAD originates from lysine decarboxylation. *ldcc* is a key gene (encoding lysine decarboxylase) in the decarboxylation of lysine for the synthesis of CAD. According to Table 1, *ldcc* was downregulated in the IP-H group compared to the WP-H group for *H. alvei* H4, while it was not activated in *P. fluorescens* ATCC13525 of the IP-P group compared to the WP-P group, which may inhibit the decarboxylation of lysine to produce CAD. The synthesis of PUT involves a relatively complex multi-step process [52], which is synthesized via ornithine decarboxylation or agmatine deamination. In the IP-H group, there was a notable decrease in the expression of three genes involved in the PUT synthesis pathway: *speA*, *speB*, and *speF*. Specifically, *speA* encodes arginine decarboxylase, *speB* encodes agmatinase, and *speF* encodes ornithine decarboxylase (Table 1), whereas no change occurred in *P. fluorescens* ATCC 13525 of the IP-P group compared to the WP-P group. The downregulation of these genes suggests a hindered process in the synthesis of PUT. On the contrary, the expression of *puuABCD* in the putrescine degradation pathway was upregulated in *H. alvei* H4 Δ*luxI* (Table 1), which accelerated the consumption rate of PUT. In addition, several pot genes encoding PUT transporter protein were downregulated in Table 1. The decrease in putrescine content was attributed to a faster rate of putrescine consumption due to increased expression of *puuABCD* and a lower rate of putrescine synthesis due to the downregulation of *speABF*. The content of cadaverine in the later stage was higher than that of putrescine (Figure 2), possibly because the anabolic pathway of cadaverine was relatively simple.

In addition, a high increase in amino acid metabolism may enable bacterial cells to adapt the structure and function of biofilm in response to environmental changes. [46]. It has also been found that amino acid biosynthesis is essential for biofilm formation [53]. In the transcription results for *P. fluorescens* ACTT13525 in the IP-P group, *CPH89_RS06815* and *katB*, which are responsible for encoding peroxidase HPII involved in tryptophan metabolism, were upregulated (Table 2). It is suspected that the metabolism of tryptophan is accelerated, leading to a decrease in biofilm content in the deletion coculture system. Meanwhile, the metabolisms of other amino acids, including threonine and D-amino-acid were dysregulated when cocultured with *H. alvei* H4 Δ*luxI*. The genes *CPH89_RS00185*, encoding aspartate/tyrosine/aromatic aminotransferase, and *CPH89_RS08005*, encoding D-amino acid dehydrogenase, were upregulated. In contrast, *CPH89_RS21450*, encoding an aspartate aminotransferase family protein involved in the biosynthesis of arginine, an essential source of protein and nucleic acid biosynthesis, is downregulated in the IP-P group. This suggests that when the QS system of *H. alvei* H4 WT in the coculture system was frustrated, an imbalanced state and disturbance of the amino acid metabolism in the cells were formed. Combined with the above considerations, QS of *H. alvei* H4 WT in the coculture affected the amino acid metabolism of *P. fluorescens* ACTT13525 at the transcriptional level.

When food is contaminated by microorganisms, the microorganisms will metabolize amino acids in proteins to produce biogenic amines. Appropriate measures need to be taken to control the content of biogenic amines in food. And the absence of QS in a coculture inhibits the production of biogenic amines in mixed bacteria by regulating amino acid metabolism, which provides ideas to inhibit the production of biogenic amines in real food processing environments.

### 3.3. Real-Time Quantitative PCR Validation (RT-qPCR)

**Figure 4:**
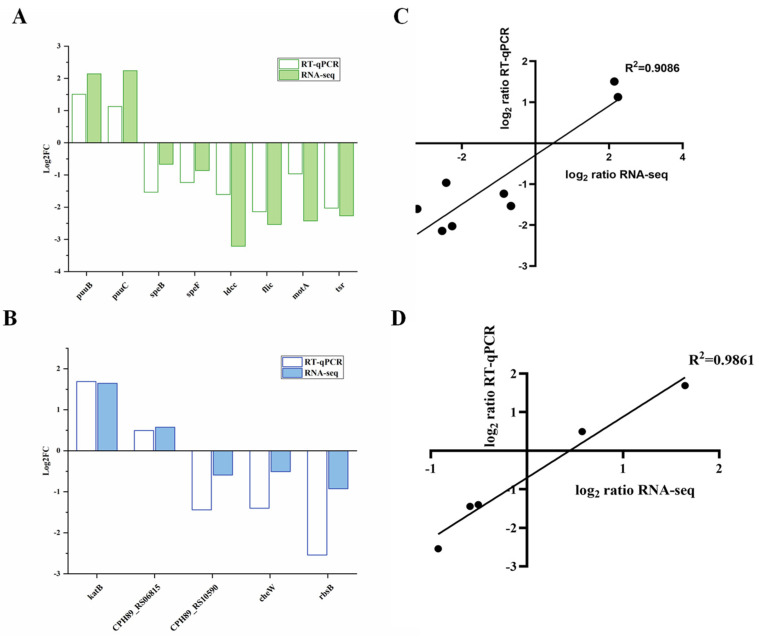
Validation of the selected DEG expression of *Hafnia alvei* H4 and *Pseudomonas fluorescens* ATCC13525 in dual species. (**A**) Quantitative reverse transcription PCR (RT-qPCR) of differentially expressed genes of *H. alvei* H4. (**B**) RT-qPCR of differentially expressed genes of *P. fluorescens* ATCC13525. (**C**) The regression line of RNA-Seq data (X-axis) plotted against the RT-qPCR data (Y-axis) of *H. alvei* H4. (**D**) The regression line of RNA-Seq data (X-axis) plotted against the RT-qPCR data (Y-axis) of *P. fluorescens* ATCC13525.

In order to confirm the validity of the expression of several genes associated with biofilm formation and spoilage obtained by RNA-seq, a total of 13 genes, including *fliC*, *motA*, *tsr*, *puuB*, *puuC*, *speB*, *speF*, and *ldcc* from *H. alvei* H4, and 5 genes belonging to various metabolic pathways (*cheW*, *katB*, *CPH89-RS06815*, *CPH89-RS10590*, and *RbsB*) from *P. fluorescens* ACTT13525 were selected for transcription polymerase chain reaction analysis (Figure 4). In *H. alvei* H4 Δ*luxI*, the gene belonging to the flagellar component (*fliC*) and bacterial chemotaxis-related genes, including methyl-accepting chemotaxis protein (*tsr*) and flagellar motor protein (*motA*), were significantly downregulated in the dual species. And in *P. fluorescens* ACTT13525 cocultured with *H. alvei* H4 Δ*luxI*, the *cheW* (related to bacterial chemotaxis) and *RbsB* (related to ABC transporters) were also downregulated. These results indicate that flagella and chemotactic-related genes are, indeed, downregulated after QS of *H. alvei* H4 WT is destroyed, thus affecting the formation of biofilm in the coculture system. In addition, three genes (*speA*, *speB*, and *speF*) within the PUT synthesis pathway also exhibited downregulation, while the expressions of *puuB* and *puuC* involved in the putrescine degradation pathway of *H. alvei* H4 were upregulated in the IP-H group compared to the WP-H group. This proves that when QS of *H. alvei* H4 WT is disrupted, the gene expression of the PUT synthesis pathway is inhibited, while the gene expression of the degradation pathway is promoted. Furthermore, the expression levels of *CPH89_RS06815* and *katB* involved in the tryptophan metabolism of *P. fluorescens* ACTT13525 were upregulated, while the gene *CPH89_RS10590* (related to amino acid metabolism) was downregulated, which suggests that QS of *H. alvei* induces metabolic disorders in *P. fluorescens*. Moreover, the results of the study indicate that the patterns of gene expression obtained from both dual RNA-seq and RT-qPCR were generally consistent, which shows that the RNA-Seq data are credible and reproducible. Additionally, a strong correlation was observed between the two data sets for each species (*H. alvei* H4 and *P. fluorescens* ATCC13525), with Pearson’s correlation coefficients of 0.9086 (Figure 4C) and 0.9861 (Figure 4D), respectively, (*p* < 0.02). This further supports the validity of the RNA-Seq data.

## 4. Conclusions

**Figure 5:**
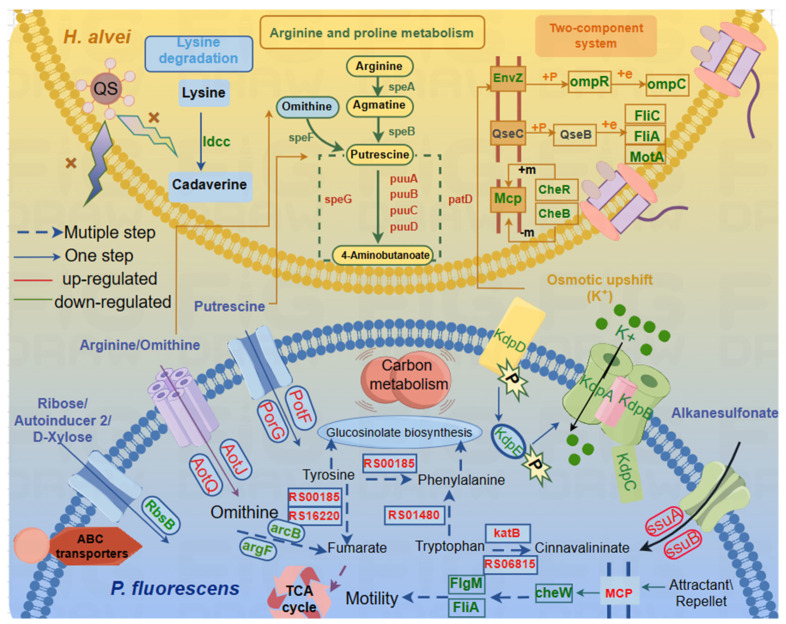
Schematic representation of certain biological pathways in *H. alvei* H4 and *P. fluorescens* affected by QS of *H. alvei* in a coculture system. This model represents the transcriptional response of genes with significant differences in transcription levels (flagellar assembly, bacterial chemotaxis, lysine degradation, arginine and proline metabolism, and two-component system) of *H. alvei* H4 and (ABC transporters, bacterial chemotaxis, phenylalanine, tyrosine, and tryptophan biosynthesis) of *P. fluorescens* ATCC13525 after the *luxI* gene knockout from *H. alvei* H4 in dual species. The yellow gradient background area represents *H. alvei* H4, and the blue gradient background area represents *P. fluorescens* ATCC13525. Most DEGs were integrated and are indicated in red (upregulated), and green (downregulated), respectively.

Overall, all the results show that QS of *H. alvei* not only regulated its own genes but also promoted microbial communication and spoilage by influencing gene expression in cocultured strains. That is, the effects of coexistence are “two-way”, where the presence of *H. alvei* affects the metabolism and behavior of *P. fluorescens* and vice versa. It was further established that QS of *H. alvei* H4 WT plays an important role in a coculture system. Based on phenotyping experiments (Figure 1), we mainly analyzed flagellar assembly, bacterial chemotaxis and the two-component system, the ABC transporter system and amino acid metabolism pathways. The schematic is shown in Figure 5. All of them are directly or indirectly related to biofilm and biogenic amine formation [54]. Specifically, the disruption of QS in *H. alvei* H4 WT inhibits the flagellar composition of *H. alvei* and the expression of the flagellar transcriptional regulators of *P. fluorescens*. At the same time, the expression of chemotactic genes in the two strains is also inhibited, thereby reducing biofilm formation. In addition, the regulation of the signaling systems is also a contributing factor to the decrease in biofilms. This includes the regulation of QS of *H. alvei* H4 WT through its two-component system, as well as the regulation of the ABC transporter of *P. fluorescens*. The reduction in the accumulation of biogenic amines is primarily caused by the downregulation of certain amino acid decarboxylase enzymes. In addition, QS of *H. alvei* H4 WT will result in an imbalance of amino acids and metabolic disorders in *P. fluorescens*, consequently impacting phenotypic changes in a coculture. This means that *H. alvei* H4 and *P. fluorescens* ATCC13525 undergo transcriptional changes in their interactions, which depend on the production of QS of *H. alvei* H4 WT. Disrupting QS of *H. alvei* H4 WT will inevitably reduce the capacity for spoilage caused by both *H. alvei* H4 WT and *P. fluorescens* ATCC13525. It indicated that QS of *H. alvei* H4 WT might offer a new target to reduce the food spoilage development caused by *H. alvei* and *P. fluorescens*. It is possible to regulate the expression of spoilage indicators such as biofilm and biogenic amines in the entire coculture system by interfering with QS. More rigorous studies are necessary to further explore the role of *H. alvei* quorum sensing in coculture regulation at the protein level.

## Figures and Tables

**Table 1:** Validation of selected DEGs related to flagellar assembly, bacterial chemotaxis, BA metabolism, and two-component system in *H. alvei* H4 (IP-H vs. WP-H).

Gene Name	log2 Radio	Gene Description
flagellar assembly		
*fliL*	−4.32515228	flagellar basal body-associated protein FliL
*fliN*	−5.766718961	flagellar motor switch protein FliN
*fliM*	−2.837097495	flagellar motor switch protein FliM
*fliJ*	−2.408896556	flagellar export protein FliJ
*ERL64_RS17280*	−1.339639572	flagellar hook-length control protein FliK
*fliS*	−2.016760592	flagellar export chaperone FliS
*motA*	−2.425518042	flagellar motor stator protein MotA
*flgB*	−3.035717278	flagellar basal body rod protein FlgB
*flgC*	−3.475398667	flagellar basal body rod protein FlgC
*flgD*	−3.258609823	flagellar hook assembly protein FlgD
*flgE*	−3.074676572	flagellar hook protein FlgE
*ERL64_RS17360*	−3.436893106	flagellar basal body rod protein FlgF
*flgG*	−3.336673997	flagellar basal body rod protein FlgG
*flgK*	−1.888983693	flagellar hook-associated protein FlgK
*flgL*	−2.226392467	flagellar hook-associated protein FlgL
*ERL64_RS17345*	−1.831507544	flagellar basal body P-ring protein FlgI
bacterial chemotaxis		
*ERL64_RS14790*	−2.265739966	methyl-accepting chemotaxis protein
*Tsr*	−2.265395717	methyl-accepting chemotaxis protein
*ERL64_RS14800*	−2.424604914	chemotaxis response regulator protein
*cheR*	−2.098189509	protein-glutamate O-methyltransferase CheR
*ERL64_RS14800*	−2.424604914	chemotaxis response regulator protein-glutamate methylesterase
two-component system		
*envZ*	−0.966750293	two-component system sensor histidine kinase EnvZ
*ompR*	−0.459688448	two-component system response regulator OmpR
*phoR*	−1.146705289	phosphate regulon sensor histidine kinase PhoR
*phoQ*	0.940798155	two-component system sensor histidine kinase PhoQ
*phoP*	1.035332663	two-component system response regulator PhoP
*rpoN*	−1.164441481	RNA polymerase factor sigma-54
BA metabolism		
*ldcc*	−3.212727378	lysine decarboxylase
*speF*	−0.860138669	ornithine decarboxylase SpeF
*speA*	−0.82353036	biosynthetic arginine decarboxylase
*speB*	−0.669157809	agmatinase
*puuA*	3.06324221	glutamine synthetase family protein
*puuB*	2.140552542	FAD-binding oxidoreductase
*puuC*	2.239399155	aldehyde dehydrogenase PuuC
*puuD*	2.663951099	gamma-glutamyl-gamma-aminobutyrate hydrolase
*patD*	1.429802723	amino butyraldehyde dehydrogenase
*ERL64_RS21280*	3.401803121	amma-amino butyraldehyde dehydrogenase
*ERL64_RS02325*	3.06324221	glutamine synthetase family protein
*speG*	0.419542916	spermidine N1-acetyltransferase
*potF*	−0.987904791	spermidine/putrescine ABC transporter substrate-binding protein PotF
*potI*	−2.362303979	putrescine ABC transporter permease PotI
*potH*	−2.568324997	putrescine ABC transporter permease PotH
*potG*	−0.987904791	putrescine ABC transporter ATP-binding subunit PotG

**Table 2:** Selected DEGs related to biofilm formation, amino acid metabolism, and ABC transport in *P. fluorescens* ATCC13525 (IP-P vs. WP-P).

Gene Name	log2 Radio	Gene Description
biofilm formation		
*fliL*	−0.925171985	flagellar basal body-associated protein FliL
*flgM*	−0.699499627	flagellar biosynthesis anti-sigma factor FlgM
*fliA*	−0.523949137	RNA polymerase sigma factor FliA
*CPH89_RS16845*	−1.029022096	OmpA family protein
*cheW*	−0.506899146	chemotaxis protein CheW
*CPH89_RS23015*	−0.547698862	methyl-accepting chemotaxis protein
*tssC*	−1.370116898	type VI secretion system contractile sheath large subunit
*tssG*	2.543096874	type VI secretion system baseplate subunit TssG
*tssH*	0.939045148	type VI secretion system ATPase TssH
*CPH89_RS09505*	1.808730303	type VI secretion system tip protein VgrG
amino acid metabolism		
*CPH89_RS22860*	−1.373666561	methionine gamma-lyase
*CPH89_RS03900*	−1.088510925	L-serine ammonia-lyase
*CPH89_RS10590*	−0.592860749	homoserine kinase
*mmsB*	−1.524151588	3-hydroxyisobutyrate dehydrogenase
*CPH89_RS06815*	0.574998597	catalase
*katB*	1.644147633	catalase KatB
*CPH89_RS08005*	2.317725314	D-amino acid dehydrogenase
*CPH89_RS00185*	0.475801482	aspartate/tyrosine/aromatic aminotransferase
*CPH89_RS21450*	1.152619749	aspartate aminotransferase family protein
*CPH89_RS16850*	0.716263227	argininosuccinate synthase
*CPH89_RS21350*	0.422007256	glutamine synthetase family protein
*gltB*	0.609812179	glutamate synthase large subunit
*CPH89_RS00200*	0.920911912	aldehyde dehydrogenase family protein
*CPH89_RS27825*	1.897595222	CoA-acylating methylmalonate-semialdehyde dehydrogenase
ABC transport		
*RbsB*	−0.923885631	sugar ABC transporter substrate-binding protein
*CPH89_RS28860*	−1.034779256	ABC transporter ATP-binding protein
*CPH89_RS10545*	−0.845831876	MetQ/NlpA family ABC transporter substrate-binding protein
*CPH89_RS24050*	−2.801510174	ABC transporter permease
*CPH89_RS19375*	−1.480685108	ABC transporter ATP-binding protein
*CPH89_RS16095*	−1.459658475	amino acid ABC transporter permease
*CPH89_RS23690*	2.876670285	carbohydrate ABC transporter permease
*urtD*	2.872150064	urea ABC transporter ATP-binding protein UrtD
*CPH89_RS08665*	1.314199994	sulfonate ABC transporter substrate-binding protein
*ssuB*	0.972531529	aliphatic sulfonates ABC transporter ATP-binding protein
*CPH89_RS15185*	0.63533128	glycine betaine ABC transporter substrate-binding protein

## Data Availability

The data presented in this study are available on request from the corresponding author. The data are not publicly available due to the need for the first author the apply for a Master’s degree.
